# Does the Length of Elbow Flexors and Visual Feedback Have Effect on Accuracy of Isometric Muscle Contraction in Men after Stroke?

**DOI:** 10.1155/2016/7641705

**Published:** 2016-03-02

**Authors:** Vilma Juodzbaliene, Tomas Darbutas, Albertas Skurvydas, Marius Brazaitis

**Affiliations:** ^1^Faculty of Sports Biomedicine, Lithuanian Sports University, Sporto Street 6, LT-44221 Kaunas, Lithuania; ^2^St. Ignatius of Loyola College, J. Jablonskio Street 2, LT-4428 Kaunas, Lithuania

## Abstract

The aim of the study was to determine the effect of different muscle length and visual feedback information (VFI) on accuracy of isometric contraction of elbow flexors in men after an ischemic stroke (IS).* Materials and Methods*. Maximum voluntary muscle contraction force (MVMCF) and accurate determinate muscle force (20% of MVMCF) developed during an isometric contraction of elbow flexors in 90° and 60° of elbow flexion were measured by an isokinetic dynamometer in healthy subjects (MH, *n* = 20) and subjects after an IS during their postrehabilitation period (MS, *n* = 20).* Results*. In order to evaluate the accuracy of the isometric contraction of the elbow flexors absolute errors were calculated. The absolute errors provided information about the difference between determinate and achieved muscle force.* Conclusions*. There is a tendency that greater absolute errors generating determinate force are made by MH and MS subjects in case of a greater elbow flexors length despite presence of VFI. Absolute errors also increase in both groups in case of a greater elbow flexors length without VFI. MS subjects make greater absolute errors generating determinate force without VFI in comparison with MH in shorter elbow flexors length.

## 1. Introduction

Stroke is one of the most frequent diseases in the world, which results in limited mobility due to impaired control of movements [1]. Stroke mostly affects muscles of one arm and leg and one side of the face (about 80% of patients) since their function largely depends on the motor cortex [1, 2]. After three months since the onset of stroke only 20% of the patients regain their normal hand function [3].

Visual feedback is extremely important when performing movements, especially the new ones, and appropriate choice of this feedback can facilitate learning of those movements [4, 5]. The feedback can be obtained from many sources: brain, muscles, tendons, skin, eyes, and so forth [6]. When the task has to be performed without visual feedback information, the motor program controlling the movement performance and regulating the perception of the movement plays a major role [7] since sensory noise, which influences the accuracy of the movement, appears when the feedback sources, for example, sight, decrease [8].

Elderly people trying to perform the movements as accurately as possible without visual feedback make errors [9, 10] and it is not clear what errors are made by men after an ischemic stroke in different muscle length.

We hypothesize that healthy men make smaller errors without visual feedback information developing 20% of their maximum voluntary muscle contraction force in comparison with men after an ischemic stroke, and the errors are even greater in both groups in case of a greater muscle length.

The aim of the study was to determine the effect of visual feedback on accuracy of isometric contraction of elbow flexors in men after an ischemic stroke at 20% of maximum voluntary muscle contraction force in different muscle length.

## 2. Materials and Methods

The study was conducted in accordance with the Declaration of Helsinki (1964). The permission (number BE-2-72) for the study was obtained from Kaunas Regional Ethics Committee for Biomedical Research.

### 2.1. The Subjects

The healthy right-handed men (MH group) (*n* = 20; age, 66.05 ± 6.2 years) and right-handed men after an ischemic stroke (MS group) during their postrehabilitation period (*n* = 20; age, 68.6 ± 6.4 years) voluntarily participated in the study. The subjects were randomly assigned to either group.

All the subjects had to comply with the inclusion criteria: (1) ischemic stroke in the middle cerebral artery basin, which occurred not earlier than one year before trial, with the left limb hemiparesis, confirmed by medical documents; (2) men over 60; (3) the subjects should not have hearing disorders which could hinder performance of the tasks; (4) the subjects should not have vision disorders which could hinder performance of the tasks; (5) the subjects should not have any other diseases of the central and peripheral nervous system (Parkinson's disease, multiple sclerosis, mental disorders, brain or medullary tumors, epilepsy, etc.); (6) the subjects should not have any endoprosthesis in the arm; (7) MMSE score should not be lower than 25; (8) muscle spasticity score according to the Ashworth scale should be 0; (9) muscle strength score according to Oxford Grading Scale should not be lower than 3; (10) balance score according to the Berg scale should not be lower than 41; (11) Barthel index score should not be lower than 91.

The subjects were acquainted with the procedure of the experiment and instructed how to perform the task correctly three days prior to the experiment.

### 2.2. Measurement of Maximum Voluntary Muscle Contraction Force (MVMCF)

Isokinetic dynamometer “Biodex System Pro 3” (Biodex Medical Systems, NY, and the computer software program version 3.29) was used for the research. The subject was seated in chair of the dynamometer (at a backrest angle of 90°). The anatomical axis of rotation was aligned to the dynamometer axis. The shoulder was abducted 15° in the frontal plane and flexed 45° in the sagittal plane. During the test, the subject held a special handle with the tested hand. The range of motion of the elbow (with the extended and flexed arm) was measured. Isokinetic and isometric muscle strength is tested in different muscle length [11]; thus the tested elbow was flexed at an angle of 90° or 60° and additionally fixed. In order to evaluate maximum voluntary muscle contraction force (MVMCF, defined as the highest peak torque, Nm), the subject had to achieve maximum voluntary force of muscle contraction. Two attempts were allowed at the angles of 90° and 60°, and the better result was registered for each angle. Short muscle length is related to weaker isometric contraction of the muscles after stroke [12]. In order to avoid extremes and to establish whether intermediate length of elbow flexors has effect on accuracy of isometric contraction in men after stroke the above-mentioned angles of the elbow were chosen.

### 2.3. Measurement of Accuracy of Elbow Flexors Isometric Contraction

20% of MVMCF was calculated individually for each subject from the force developed by them. The subjects had to perform two isometric contractions of the elbow flexors with visual feedback and one attempt without it. Every contraction of a muscle lasted for 15 s with intervals of 10 s (Table 1). The discretization of the signal was 10 ms.

In order to obtain accurate results of MVMCF, only the middle 10 s interval of 15 s was taken into consideration since other intervals of the signal represented the contraction of the muscles (2.5 s at the beginning) and possible fatigue (2.5 s at the end). When the subjects had to perform the task with VFI, they saw the indicatory line of their 20% of MVMCF on the screen of the isometric dynamometer, which provided them with the information about the generated force. The task was performed with the elbow flexed at 90° and 60° with a 5-minute break between the tasks.

In order to evaluate the accuracy of the isometric contraction of the muscle, absolute errors were calculated. The absolute errors provided information about the difference between determinate and achieved force and showed the accuracy of the isometric contraction [13]. They were calculated according to the formula:

(1) $$\begin{array}{c} \text{Absolute error}=\frac{\sum \left|x_{i}-T\right|}{n}, \end{array}$$

where *x*_*i*_ is mean force (Nm) developed in the 10 s interval; *T* is 20% of MVMCF (Nm); *n* is number of attempts; value inside vertical brackets (|·|) is considered positive.

Absolute errors showed absolute deviation from the determinate contraction force of the muscle. For example, if the subject needs to exert efforts in order to perform an isometric contraction of the muscles at 20% of MVMCF, which makes 10 Nm, and he/she performs at 8.5 Nm, the absolute deviation equals 1.5 Nm; if the subject performs at 11 Nm, the absolute deviation equals 1 Nm.

### 2.4. Statistical Analysis

Data are reported as means ± SD within the text and they are displayed as means ± SE in the figures. The data were tested for normal distribution using the Kolmogorov-Smirnov test, and all data were found to be normally distributed. Parametric paired *t*-test was used to test for differences between muscle force data in respect to different muscle length and VFI. Independent samples *t*-test was used to test for differences between variables in MH and MS groups. One sample *t*-test was used to determine differences between muscle force (%) and target force. The level of significance was set at *P* < 0.05 and all statistical analyses were performed using IBM SPSS Statistics 22 (IBM Corporation, Armonk, NY).

## 3. Results

MVMCF of elbow flexors of both arms was tested at the beginning of the experiment. 20% of MVMCF was calculated individually for each subject. Subjects had to perform tasks according to calculated MVMCF value with VFI and without it.

The average MVMCF of the elbow flexors of the right arm of healthy men was 58.24 ± 5.6 Nm and greater than in men after an ischemic stroke 49.13 ± 11.4 Nm (*P* = 0.04) with elbow flexed at 90°. The average MVMCF of the left arm of healthy men was 56.48 ± 9.9 Nm and greater than in men after an ischemic stroke 46.53 ± 9 Nm (*P* = 0.02) with elbow flexed at 90°.

Analogous results were obtained developing MVMCF with elbow flexed at 60°. The average MVMCF of the elbow flexors of the right arm of healthy men was 60.46 ± 10.1 Nm and greater than in men after an ischemic stroke 46.2 ± 11.6 Nm (*P* = 0.006) with elbow flexed at 60°. The average MVMCF of the left arm of healthy men was 61.96 ± 11.2 Nm and greater than in men after an ischemic stroke 51.11 ± 13.6 Nm (*P* = 0.03) with elbow flexed at 60°.

In order to achieve 20% of MVMCF with elbow flexed at 90° healthy men had to perform 11.65 ± 1.1 Nm of isometric contraction of elbow flexors with the right arm and 11.29 ± 1.9 Nm with the left arm; meanwhile, the men after an ischemic stroke had to achieve 9.83 ± 2.3 Nm with the right arm and 9.31 ± 1.8 Nm with the left arm.

In order to achieve 20% of MVMCF with elbow flexed at 60° healthy men had to perform 12.09 ± 2 Nm of isometric contraction of elbow flexors with the right arm and 12.39 ± 2.2 Nm with the left arm; and the men after an ischemic stroke had to achieve 9.24 ± 2.3 Nm with the right arm and 10.22 ± 2.7 Nm with the left arm.

**Figure 1:**
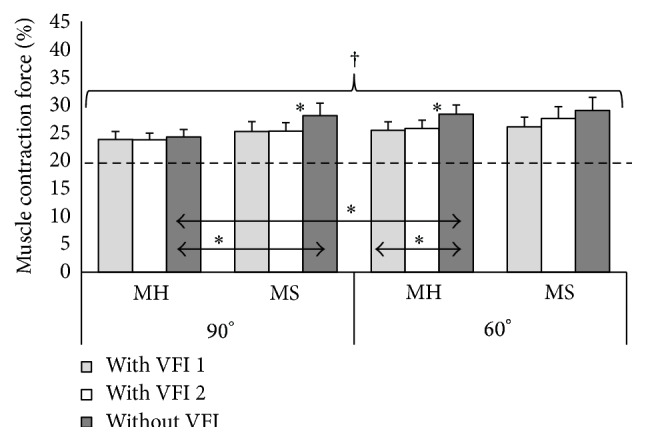
Isometric muscle contraction force of the right elbow flexors in respect of 20% of maximum voluntary muscle contraction force with elbow flexed at 90° and 60°. ^*∗*^ *P* < 0.05; ^†^ *P* < 0.05, significantly different from the target force (20%); MH: healthy men group; MS: men after ischemic stroke group; VFI: visual feedback information.

**Figure 2:**
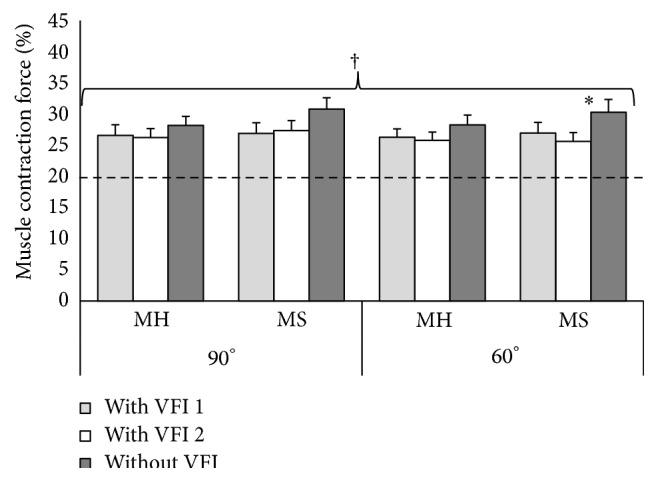
Isometric muscle contraction force of the left elbow flexors in respect of 20% of maximum voluntary muscle contraction force with elbow flexed at 90° and 60°. ^*∗*^ *P* < 0.05; ^†^ *P* < 0.05, significantly different from the target force (20%); MH: healthy men group; MS: men after ischemic stroke group; VFI: visual feedback information.

**Figure 3:**
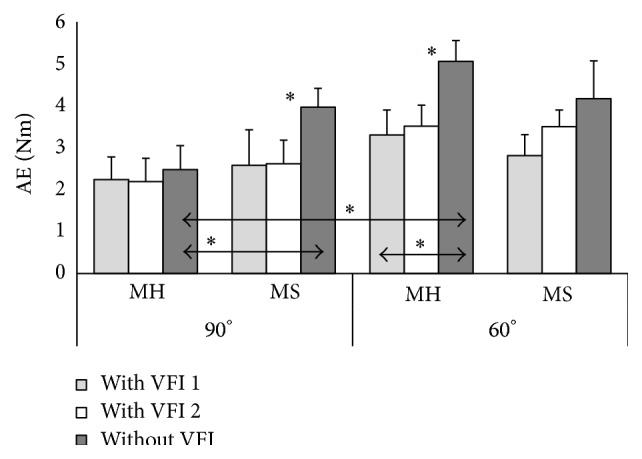
Absolute errors of the right elbow flexors isometric contraction at 20% of maximum voluntary muscle contraction force with elbow flexed at 90° and 60°. ^*∗*^ *P* < 0.05; MH: healthy men group; MS: men after ischemic stroke group; AE: absolute errors; VFI: visual feedback information.

**Figure 4:**
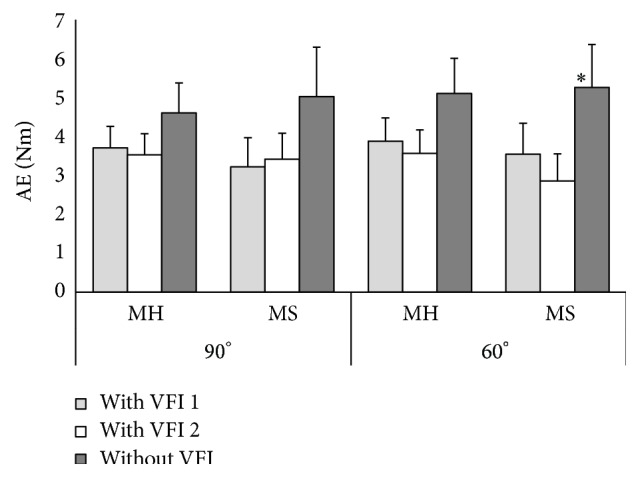
Absolute errors of the left elbow flexors isometric contraction at 20% of maximum voluntary muscle contraction force with elbow flexed at 90° and 60°. ^*∗*^ *P* < 0.05; MH: healthy men group; MS: men after ischemic stroke group; AE: absolute errors; VFI: visual feedback information.

The accuracy of voluntary isometric muscle contraction force of the elbow flexors was estimated according to generated muscle force in respect to 20% of MVMCF (Figures 1 and 2) and absolute errors representing absolute deviation from the determinate contraction force (Figures 3 and 4). Stroke survivors had to achieve relatively smaller determinate isometric muscle contraction force in comparison with healthy subjects. Despite above-mentioned fact healthy men and men after stroke produced inaccurate determinate force of elbow flexors of both arms in different muscle length (Figures 1 and 2). Achieved isometric contraction force significantly differed from target force (*P* < 0.01).

Subjects of MH group had to perform the task with the right arm flexed at 90° with VFI during the first attempt and they made an error by 2.24 ± 0.54 Nm; meanwhile, subjects of MS group made an error by 2.58 ± 0.85 Nm (*P* = 0.36) (Figure 3). Similar results were obtained during the second attempt with VFI: subjects of MH group made an error by 2.19 ± 0.56 Nm and subjects of MS group by 2.62 ± 0.56 Nm (*P* = 0.15). The errors of MH group were smaller 2.48 ± 0.57 Nm than of MS group 3.97 ± 0.45 Nm when the same task was performed with the right arm without VFI (*P* = 0.03). Statistically significant differences were obtained when comparing results of MS group with and without VFI during their second attempt (*P* = 0.04).

Subjects of MH group had to perform the task with the right arm flexed at 60° with VFI during their first attempt and they made an error by 3.3 ± 0.6 Nm and subjects of MS group made an error by 2.81 ± 0.5 Nm (*P* = 0.28) (Figure 3). The results of the second attempt with VFI were the following: subjects of MH group made an error by 3.51 ± 0.5 Nm and subjects of MS group by 3.5 ± 0.4 Nm (*P* = 0.48). When subjects had to perform the same task with their right arm without VFI MH group made greater error 5.06 ± 0.5 Nm compared to error of first attempt with VFI (*P* = 0.03) and second attempt with VFI (*P* = 0.04). There were no statistically significant differences between MS group errors with and without VFI 4.17 ± 0.9 Nm.

MH group made significantly greater errors generating determinate muscle force with the right arm flexed at 60° (5.06 ± 3.35 Nm), that is, in greater muscle length in comparison with shorter muscle length (2.48 ± 1.8 Nm) (*P* = 0.02).

The healthy and poststroke subjects made the errors of similar value while performing the task with left arm flexed at 90° with and without WFI (Figure 4). Subjects of MH group during their first attempt with VFI made an error by 3.71 ± 0.55 Nm and subjects of MS group made an error by 3.22 ± 0.75 Nm (*P* = 0.30). The results of the second attempt with VFI were the following: subjects of MH group made an error by 3.53 ± 0.54 Nm and subjects of MS group by 3.42 ± 0.67 Nm (*P* = 0.45). Subjects of MH group performing the same task with their left arm without VFI made an error by 4.61 ± 0.77 Nm and subjects of MS group by 5.03 ± 1.2 Nm (*P* = 0.38).

The subjects of MH group had to perform the task with the left arm flexed at 60° with VFI during their first attempt and made an error by 3.88 ± 0.6 Nm and subjects of MS group by 3.54 ± 0.8 Nm (*P* = 0.38) (Figure 4). The results of the second attempt with VFI were the following: subjects of MH group made an error by 3.57 ± 0.6 Nm and subjects of MS group by 2.86 ± 0.7 Nm (*P* = 0.24). When subjects had to perform the same task with their left arm without VFI, the results were the following: subjects of MH group made an error by 5.11 ± 0.9 Nm and subjects of MS group by 5.27 ± 1.1 Nm (*P* = 0.46).

In analysis of the results of both groups with respect to a flexor muscle length, that is, angle of elbow flexion, the tendency to make greater absolute errors in greater muscle length without VFI was noticed. The similar tendency was noticed analyzing results of the second attempt with VFI. During the attempt without VFI, greater errors were performed with the right (dominant) arm with elbow flexed at 60° (5.06 ± 0.5 Nm) compared to 90° (2.48 ± 0.57 Nm) (*P* = 0.002) or compared to errors made with VFI (3.51 ± 0.6 Nm) (*P* = 0.03) in the MH group.

## 4. Discussion

The goal of the study was to determine the effect of visual feedback on accuracy of the contraction of elbow flexors in different muscle length in stroke survivors. The MVMCF, generated target force, and errors made achieving the determinate muscle force were estimated.

MVMCF of both arms in men after stroke was weaker than in healthy men despite elbow flexors length. It can depend on the fact that the number of motor neurons and their pulsation frequency decrease and intramuscular coordination deteriorates after an ischemic stroke; when normal activation is absent, the muscle atrophies and shortens, amount of connective tissue increases, and muscle becomes less plastic. These are the major causes for the muscle MVMCF to decrease after an ischemic stroke [14].

Also significant differences of the muscle force have been established between the healthy and impaired arm as well as between the healthy and impaired leg in stroke survivors [15].

We can make assumption that elbow flexors of stroke survivors were weaker in comparison to healthy subjects due to changes in muscle properties, alteration in motor unit activation, and motor unit recruitment in disorder as it was stated by other authors [16].

The analysis of the accuracy of generated muscle force in respect to target force revealed that healthy men and stroke survivors produced inaccurate force of elbow flexors of both arms in different muscle length with different visual feedback. The age of the subjects as well as intrinsic muscle properties after stroke could have the significant impact on these results [17].

When the visual feedback was present healthy and poststroke subjects made similar errors generating determinate muscle force with right (dominant) arm regardless of muscle length.

The healthy men and men after stroke made significantly greater absolute errors performing task without VFI. The stroke survivors made greater absolute errors in comparison to healthy men performing the task with the right arm with the elbow flexed at 90° without VFI. It can be explained that the movement of the right (dominant) arm without VFI results in greater absolute errors as the neuromuscular system cannot obtain sight-based information, which helps make movement corrections [18]. The VFI affects accuracy of the performed task not only in stroke survivors, but also in the healthy persons. These results confirm earlier findings that when the movement is performed with external feedback information the subject can easily control the motion observing the movement trajectory; however, with external feedback eliminated, only internal feedback can be trusted [19]. The internal feedback as well as the perception of the generated force might be altered in stroke survivors as it was reported in other studies also [20].

The tendency to make greater absolute errors regardless of muscle length was noticed when the task was performed without VFI. It is clear that the subject could more accurately perform an isometric contraction of muscles upon seeing the force line on the screen. It is believed that performing the task with VFI the right lower and the front lobe of the cortex are activated, which helps correct the movement performance [21]. Thus, VFI provided a possibility to perform the necessary movement more accurately. Other researchers agree on this and claim that the learning process also gets more complicated without feedback information [5, 22].

We had established that the errors made by healthy subjects and stroke survivors achieving determinate force were similar when the task was performed with the left arm. In the present study, we confirmed previous work. According to the other authors, the obtained results showed that the right brain hemisphere was oriented towards accuracy; thus, the results for the left arm were similar in both groups [23].

Some researchers claim that the movement is performed with a more accurate trajectory and greater accuracy in greater muscle length [24, 25]. According to this statement the results obtained in the present study could appear contradicting as it turned out that when the task was performed with the right (dominant) arm and without VFI absolute errors were greater in case of a greater muscle length in comparison with the shorter muscle length. The same tendency was observed when the task was performed with the right arm with VFI. Healthy subjects and stroke survivors had to generate muscle force instead of making the movement; thus, the results of our study should be analyzed in respect of force-length properties of muscles. This could explain why accuracy of isometric contraction in greater muscle length was worse in comparison with shorter muscle length. In general there were only few results revealing dependence of accuracy of isometric muscle contraction on elbow flexors length. And it was complicated to establish substantial relationship between muscle length and accuracy of isometric muscle contraction in men after stroke. Whereas visual feedback had the significant influence achieving determinate force.

## 5. Conclusions

There is a tendency that greater absolute errors generating determinate force are made by healthy and poststroke subjects groups in case of a greater elbow flexors length despite presence of visual feedback. Absolute errors also increase in both groups in case of a greater elbow flexors length without visual feedback. Men after an ischemic stroke make greater absolute errors generating determinate force without visual feedback in comparison with healthy men in shorter elbow flexors length.

## Figures and Tables

**Table 1:** Study trial protocol.

Familiarisation + MVMCF				
Rest, 1 day				
20% of MVMCF with VFI (1) elbow flexed at 90°, 15 s	Rest, 10 s	20% of MVMCF with VFI (2) elbow flexed at 90°, 15 s	Rest, 10 s	20% of MVMCF without VFI elbow flexed at 90°, 15 s

Rest, 1 minute				
20% of MVMCF with VFI (1) elbow flexed at 60°, 15 s	Rest, 10 s	20% of MVMCF with VFI (2) elbow flexed at 60°, 15 s	Rest, 10 s	20% of MVMCF without VFI elbow flexed at 60°, 15 s

MVMCF: maximum voluntary muscle contraction force, VFI: visual feedback information.
